# Dietary patterns during pregnancy in relation to maternal dietary intake: The Mutaba’ah Study

**DOI:** 10.1371/journal.pone.0312442

**Published:** 2024-10-22

**Authors:** Aisha A. Almulla, Hanna Augustin, Luai A. Ahmed, Linnea Bärebring

**Affiliations:** 1 Department of Internal Medicine and Clinical Nutrition, Institute of Medicine, Sahlgrenska Academy, University of Gothenburg, Gothenburg, Sweden; 2 Dietary Services, Tawam Hospital, Abu Dhabi Health Services Company (SEHA), Abu Dhabi, United Arab Emirates; 3 Institute of Public Health, College of Medicine and Health Sciences, United Arab Emirates University, Al Ain, United Arab Emirates; 4 Zayed Centre for Health Sciences, United Arab Emirates University, Al Ain, United Arab Emirates; Universidad Señor de Sipán, PERU

## Abstract

**Aim:**

To relate adherence to healthy dietary patterns, evaluated by different dietary indices, to the intake of nutrients and food groups among pregnant women in the United Arab Emirates.

**Methods:**

The analyses included 1122 pregnant women from the Mutaba’ah Study. Dietary intake was assessed using a semi-quantitative Food Frequency Questionnaire. Adherence to three dietary pattern indices was assessed; Alternate Healthy Eating Index for Pregnancy (AHEI-P), Alternate Mediterranean Diet (aMED) and Dietary Approaches to Stop Hypertension (DASH). Associations between adherence (score >median) to the three dietary indices and intake of nutrients and food groups were analyzed using logistic regression analysis.

**Results:**

Women with higher intake of polyunsaturated fatty acids, fiber, vegetables, fruits, legumes, and nuts and lower intake of saturated fatty acids, red meat, and sweetened beverages had significantly higher odds of adherence to all three dietary patterns (p<0.05). Associations between intakes of nutrients and food groups with odds of adherence to the dietary patterns differed for total fat (only with AHEI-P, [odds ratio [OR]: 0.96; 95% confidence interval [CI]: 0.94–0.98]) and monounsaturated fatty acids (only with aMED, [OR: 1.06; 95% CI: 1.02–1.10]), dairy (with AHEI-P [OR: 0.89; 95% CI: 0.84–0.95] and aMED [OR: 0.86; 95% CI: 0.81–0.91], and with DASH [OR: 1.10; 95% CI: 1.04–1.17]), whole grain (only with aMED [OR: 2.19; 95% CI: 1.61–2.99] and DASH [OR: 4.27; 95% CI: 3.04–5.99]) and fish (with AHEI-P [OR: 1.36; 95% CI: 1.02–1.80] and aMED [OR: 1.79; 95% CI: 1.35–2.38], and with DASH [OR: 0.67; 95% CI: 0.52–0.86]).

**Conclusion:**

Adherence to the three dietary pattern indices was generally associated with a favorable intake of nutrients and food groups. However, the indices captured slightly different aspects of dietary intake. These results show that dietary indices that assess adherence to healthy dietary patterns cannot be used interchangeably.

## Introduction

A healthy diet is important for health and nutritional status [1]. According to the World Health Organization [2], eating a variety of foods and consuming less salt, sugar, and saturated and trans-fatty acids are essential parts of a healthy diet. Generally, eating habits are of greater importance for health than intakes of single nutrients [3, 4]. Therefore, dietary patterns that reflect overall dietary habits are commonly used in nutritional research [5, 6] and may provide important information on how habitual diet is associated with diseases [7].

Various indices have been proposed to evaluate overall diet quality. The Healthy Eating Index (HEI) was developed by the United States Department of Agriculture (USDA) [8], and was revised as the Alternate Healthy Eating Index (AHEI), which better predicted the risk of cardiovascular disease and other major chronic diseases than the original HEI [9]. Another commonly used dietary index is the Mediterranean Diet Score [10], and was modified as the alternate Mediterranean Diet (aMED) score, focusing on higher consumption of plant foods, monounsaturated fat, and fish, and lower consumption of animal products and saturated fat [11, 12]. In addition, an index to evaluate adherence to the Dietary Approaches to Stop Hypertension (DASH) diet, a dietary pattern initially proposed for the reduction of blood pressure [13], have been developed [14], and is included in the 2020–2025 Dietary Guidelines for Americans as an example of a healthy eating pattern [15]. Several studies showed that the indices AHEI [16], aMED [12], and DASH [14], are inversely correlated with the risk of death from cardiovascular disease, cancer, and all-cause mortality [17–25]. Moreover, RCT studies have demonstrated that the DASH diet lowers blood pressure [17, 26, 27], and that the Mediterranean Diet lowers the risk of cardiovascular diseases [28, 29].

During pregnancy, a healthy dietary pattern decreases the risk of maternal and fetal complications and long-term negative health outcomes in the offspring [30]. Moreover, a high diet quality with a higher nutrient profile may promote healthy weight gain and prevent excessive gestational weight gain (GWG) [31], and improve fetal growth in general [32]. The Dietary Guidelines for Americans 2020–2025 highlight the importance of healthy dietary patterns during pregnancy and lactation [15, 33]. Observational studies show that dietary patterns are related to pregnancy and fetal outcomes in predominantly Western countries. For example, studies showed that adherence to healthy dietary patterns is associated with a lower risk of preterm birth [34, 35], preterm delivery [36], lower blood glucose [37], preeclampsia [35, 38], and gestational diabetes [35, 39, 40]. Dietary indices can capture the intake of nutrients and food groups in pregnant women. Among pregnant women in the US, higher scores of AHEI, aMED, or DASH were associated with healthier profile of dietary intake, reflected in for example, higher intake of vegetables, whole fruits, whole grains, and fish [35].

In the United Arab Emirates (UAE), there is minimal data on dietary patterns and adherence to dietary recommendations among pregnant women. A previous small birth cohort (n = 242) in the UAE found that an unhealthy dietary pattern (defined by a data-driven approach) was associated with higher GWG, while a healthier dietary pattern was associated with lower GWG rate [41]. To our knowledge, adherence to a priori defined healthy dietary patterns among pregnant women in the UAE has not been assessed or related to the intake of nutrients or food groups. Therefore, the present study aims to relate adherence to healthy dietary patterns, evaluated by three dietary indices (Alternate Healthy Eating Index for Pregnancy (AHEI-P), aMED and DASH), to intake of nutrients and food groups among pregnant women in the UAE.

## Materials and methods

### Study design and setting

Data used in these analyses are part of the Mutaba’ah Study [42], an ongoing prospective cohort study investigating maternal and early life determinants of maternal, infant, child, and adolescent health in the UAE. Recruitment started in May 2017 in major hospitals in Al Ain city. Inclusion criteria were pregnant women from the Emirati population who were 18 years or older, residents in Al Ain, and able to provide informed consent were eligible to be included in the study. Pregnant women with implausible reported energy intake (<600 kcal or >4000 kcal/day) were excluded from this analysis. Between the 9^th^ of December 2019 and 26^th^ of August 2022, the recruited pregnant women were also asked to provide dietary data, and the respondents formed a dietary subcohort within the Mutaba’ah Study. More detailed information about the Mutaba’ah Study has been published previously [42]. Ethical approval was obtained from the UAE University Human Research Ethics Committee (ERH-2017-5512), and the Abu Dhabi Health Research and Technology Ethics Committee (DOH/CVDC/2022/72) [42]. Informed written consent was obtained from all participants prior to data collection. All study procedures were performed according to the guidelines of the Declaration of Helsinki.

Data were obtained from medical records and self-administered questionnaires [42]. Maternal demographic characteristics were obtained from a baseline questionnaire, administered at inclusion to the Mutaba’ah Study, and included maternal age, gestational age, parity, gravidity, education level, and occupation status. Data regarding weight and height were extracted from medical records. Body mass index (BMI) was calculated (kg/m^2^) and categorized based on the criteria from the World Health Organization [43]. Parity was defined as nulliparous (never given birth) or parous (given birth once or more) [44]. Gravidity was defined as primigravid (pregnant for the first time) or multigravida (pregnant more than once) [44].

### Dietary assessment and indices

Maternal diet during pregnancy was assessed using a validated self-administered 146-item semi-quantitative Food Frequency Questionnaire (FFQ) administered at recruitment to the Mutaba’ah Study. The FFQ was answered during antenatal care visits at any time point during pregnancy and reflected habitual dietary intake during the entire pregnancy until that point. The FFQ has been validated in a subsample of the cohort [45]. In the FFQ, specified portion sizes were given for each food item, and women were asked to choose from nine possible intake frequencies, ranging from never or less than once/month to >6 times/day. For missing responses to single questions in the FFQ, we used imputation by replacing the missing value with the mean of the available responses for that item. This approach was mainly applied in the sweets and baked goods category. Further, N = 25 participants completed the FFQ twice (either during the same pregnancy or in two different pregnancies) and only the first FFQ completed by each woman was included. Intake data from the FFQ was then used to calculate the scores of the dietary indices. Three dietary indices were used to assess overall adherence to healthy dietary patterns; AHEI-P [37], aMED [10, 12], and DASH [14]. A detailed description of each score index is provided in S1 Appendix.

The AHEI-P index is a pregnancy-modified version of the AHEI [9, 37], by the exclusion of alcohol, nuts and soy protein [37], further described in S1 Appendix. The AHEI-P measures adherence to the USDA dietary recommendations on a 90-point scale with the following nine components: vegetables, fruit, ratio of white to red meat, fiber, trans fat, ratio of polyunsaturated fatty acids (PUFA) to saturated fatty acids (SFA), folate, calcium, and iron. Intakes of fruits and vegetables were calculated as servings/day by summarizing the frequencies of each contributing food item. For those consuming no red meat and those consuming no red or white meat, the ratio of white to red meat was set to 1 (the maximum score). Intakes of micronutrients were energy-adjusted using the residual method [46]. Intakes from vitamin and mineral supplements were not included. Each component of the AHEI-P contributed 0–10 points to the total score, where a score of 10 indicates maximal adherence to the recommendation, and a score of 0 indicates minimum adherence. The scores from each component were summarized to obtain the total AHEI-P score ranging from 0 (minimal possible adherence) to 90 (maximal possible adherence).

The aMED score is based on the Mediterranean Diet score [10, 12], but has been modified to fit pregnancy by excluding alcohol intake [35]. It is based on eight components: vegetables, fruits, nuts, whole grains, legumes, fish, monounsaturated fat (MUFA) to SFA ratio, and red and processed meat. The total score ranges from zero to eight, with a higher score representing higher adherence to the Mediterranean dietary pattern. The score was calculated based on the median intake for each component of the index, within the study sample. Intake equal to and above the median was scored as 1 point for healthy components (vegetables, legumes, fruits, nuts, whole grains, fish, and the ratio of MUFA:SFA), while intakes below the median received 0 points. Intake of red and processed meat was scored inversely, with an intake below the median receiving 1 point and intakes equal to and above the median receiving 0 points. Scores from each component were summarized to calculate the total aMED score (see S1 Appendix).

The DASH score, adapted for pregnancy [14, 35], consists of 8 components: fruits, vegetables, nuts and legumes, low-fat dairy products, whole grains, sodium, sugar-sweetened beverages, and red and processed meat. The total score of the DASH index ranges from 8 to 40, and a higher score represents higher adherence to the DASH dietary pattern. The components were scored on a scale of 1 to 5 based on quintiles of intake within the study sample. For intakes of healthy components (fruits, vegetables, nuts and legumes, low-fat dairy products, whole grains), intake in quintile 1 was assigned 1 point and intake in quintile 5 was assigned 5 points. Less healthy components (sodium, sugar-sweetened beverages, and red and processed meats) received the reverse scoring i.e. intake in quintile 1 was assigned 5 points and intake in quintile 5 was assigned 1 point. Then, the component scores were summarized to obtain the overall DASH score (see S1 Appendix).

### Statistical analysis

Demographic characteristics were described as means and standard deviations (SD) or medians and quartiles for continuous variables and frequencies and proportions for categorical variables. Normality was examined by histograms. The total scores of all three dietary indices were normally distributed.

Associations between the three dietary indices (AHEI-P, aMED, DASH), and maternal dietary intake (total energy (kcal) and (MJ), macronutrients (carbohydrates, protein, and fat and their subtypes) and food groups) were assessed. Firstly, the differences in nutrient intake were analyzed between tertiles of each dietary index using Kruskal-Wallis test. Secondly, correlations between the three indices were examined by Spearman’s rank correlation. To further analyze the association between the dietary intake with each dietary index, two adjusted binary logistic regression analysis models were used; 1: total energy intake, and 2: total energy intake, age, gravidity, gestational age, education level and occupation status. Each dietary index was categorized based on the median as low adherence (0: below the median) and high adherence (1: equal and above the median). Low adherence was used as the reference category. The odds ratios (OR) with 95% confidence intervals (CI) were reported. For all statistical analyses, a statistical significance level of p<0.05 was applied. All statistical analyses were performed using Statistical Package for Social Sciences version 28, IBM Corporation (IBM SPSS Statistics for Windows, version 28.0).

## Results

From the Mutaba’ah cohort study, 1531 pregnant women were enrolled in the current dietary subcohort, and 1122 women were included in this analysis after excluding those with implausible energy intake (<600 kcal/d (n = 31) or >4000 kcal/d (n = 378)).

Table 1 shows the demographic characteristics of the pregnant women included in this analysis. The mean±SD age was 31±6 years, and the mean gestational age was 6.5±2 months at inclusion in the Mutaba’ah Study, with most (58%) in the third trimester. In the first trimester, the mean BMI was 27± 6 kg/m^2^, of which 4% were underweight, 34% normal weight, 34% overweight, and 28% obese. The proportion of multigravida was 76%, and multiparity was 71%. Overall, 48% had a university level education, 53% were housewives, and 99.6% were non-smokers.

**Table 1:** Maternal demographics characteristics of 1122 pregnant women included in the analysis.

Continuous variables	N (valid values)	Mean± SD
Age, years	1122	31±6
Gestational age (months)	1122	6.5±2
First trimester BMI (kg/m^2^)	768	27±6
**Categorical variables**		**N (valid %)**
Gestational age (months)	1122	
1^st^ trimester (1–3)		131 (12)
2^nd^ trimester (4–6)		339 (30)
3^rd^ trimester (7–9)		652 (58)
First trimester BMI (kg/m^2^)	768	
Underweight <18.5		31 (4)
Normal weight 18.5–24.9		260 (34)
Overweight ≥25		260 (34)
Obese ≥30		217 (28)
Gravidity	1121	
Primigravid		268 (24)
Multigravida		853 (76)
Parity	1121	
Nulliparous		322 (29)
Multiparous		799 (71)
Education level	1051	
≤ Primary school		48 (5)
High school or diploma		496 (47)
University level		507 (48)
Occupation status	1048	
Student		84 (8)
Housewife		556 (53)
Seeking employment		100 (10)
Employed		308 (29)
Smoking	716	
Non-smoker		713 (99.6)
Smoker		3 (0.4)

BMI, Body mass index; SD, Standard deviation.

The median (Q1-Q3) AHEI-P score was 63 (58–67) (minimum 27 to maximum 85) (S1 Table). The median aMED score was 4 (3–5) (minimum 0 to maximum 8), and the median DASH score was 23 (20–25) (minimum 11 to maximum 37) (S2 Table). The three dietary indices significantly correlated with one another, with Spearman’s correlation coefficient between AHEI-P and aMED rho = 0.42 (p<0.001), AHEI-P and DASH rho = 0.56 (p<0.001) and aMED and DASH rho = 0.46 (p<0.001).

Table 2 shows the maternal dietary intake by tertiles of the three dietary indices scores. Intake of fiber, vegetables, fruits, whole grains, legumes, and nuts increased with higher tertiles of the three scores (all p<0.05). Intake of sweetened beverages decreased with higher tertiles of all three indices (p<0.01). PUFA E% increased, and SFA E% decreased significantly with higher tertiles of all the three scores (all p<0.001). However, MUFA E% increased with higher tertiles of AHEI-P only (p = 0.002). Energy intake increased with higher tertiles of AHEI-P and DASH score and decreased with higher tertiles of aMED score (p<0.001). Carbohydrate E% increased with higher tertiles of AHEI-P and aMED scores (p<0.001 and p = 0.014, respectively), but not with DASH. Moreover, dairy intake increased with higher tertiles of AHEI-P and DASH (p<0.001) but decreased with higher tertiles of aMED (p<0.001). Fish intake increased with higher tertiles of AHEI-P and aMED (p<0.001), but not with DASH. In contrast, red meat intake decreased with higher tertiles of aMED and DASH (p<0.001), but not AHEI-P.

**Table 2:** Maternal dietary intake according to the tertiles of the AHEI-P, aMED and DASH indices scores^1,2^.

Maternal dietary intake	AHEI-P				aMED				DASH				
	Tertile 1	Tertile 2	Tertile 3	P-value	Tertile 1	Tertile 2	Tertile 3	P-value	Tertile 1	Tertile 2	Tertile 3	P-value	Overall
Energy intake, kcal/d	1848±801	2238±737	2641±700	<0.001	2389±819	2199±806	2013±755	<0.001	2167±785	2151±800	2407±835	<0.001	2239±813
Energy intake MJ/d	8±3	9±3	11±3	<0.001	10±3	9±3	8±3	<0.001	9±3	9±3	10±4	<0.001	9±3
Carbohydrate, E%	47.9±9.7	50.4±8.8	49.8±7.6	<0.001	48.8±9.8	49.4±8.4	50.8±7.3	0.014	48.6±8.9	49.6±8.6	50.2±8.8	0.14	49.4±8.8
Protein, E%	16.4±3.9	16.2±3.3	16.1±2.5	0.54	16.1±3.8	16.3±3.1	16.3±2.6	0.45	16.4±3.4	16.0±3.3	16.2±3.2	0.12	16.2±3.3
Fat, E%	37.9±7.2	36.6±7.0	37.6±6.5	0.002	37.6±7.4	37.5±6.9	36.5±5.8	0.17	37.4±6.7	37.4±6.8	37.3±7.2	0.99	37.4±6.9
PUFA, E%	6.3±2.3	6.7±2.3	7.6±2.6	<0.001	6.3±2.3	7.1±2.5	7.4±2.2	<0.001	6.4±1.9	7.1±2.4	7.2±2.8	<0.001	6.9±2.4
MUFA, E%	14.6±3.2	14.5±3.5	15.3±3.4	0.002	14.5±3.4	14.9±3.5	14.8±2.9	0.26	14.6±3.2	14.8±3.3	14.9±3.6	0.56	14.8±3.4
SFA, E%	13.5±3.8	11.9±2.7	11.2±2.1	<0.001	13.2±3.7	11.9±2.5	10.8±1.9	<0.001	12.8±3.4	12.0±2.8	11.7±2.7	<0.001	12.2±3.1
Fiber, g/d	19.6±11.6	28.5±11.1	38.8±13.4	<0.001	27.4±14.1	29.4±14.5	30.7±14.2	0.03	23.9±12.1	28.3±12.9	35.7±15.4	<0.001	28.9±14.3
Vegetables, serving/d	0.7±0.6	1.3±1.2	2.6±2.4	<0.001	1.3±2.2	1.6±1.5	1.9±1.2	<0.001	1.1±1.9	1.3±1.1	2.3±1.8	<0.001	1.6±1.8
Fruits, serving/d	3.4±3.6	5.4±3.5	6.9±3.6	<0.001	5.1±4.1	5.2±3.8	5.6±3.3	0.031	4.1±3.3	5.2±3.5	6.7±4.2	<0.001	5.2±3.8
Dairy, serving/d	2.8±2.7	2.8±2.3	3.2±2.1	<0.001	3.6±2.9	2.7±1.9	2.2±1.4	<0.001	2.7±2.1	2.6±2.3	3.6±2.7	<0.001	2.9±2.4
Wholegrain, serving/d	0.4±0.5	0.4±0.5	0.5±0.6	<0.001	0.4±0.6	0.5±0.5	0.6±0.5	<0.001	0.3±0.4	0.4±0.6	0.7±0.6	<0.001	0.4±0.5
Fish, serving/d	0.4±0.5	0.6±0.7	0.6±0.6	<0.001	0.5±0.8	0.6±0.5	0.6±0.4	<0.001	0.6±0.8	0.5±0.5	0.5±0.4	0.16	0.5±0.6
Red meat, serving/d	0.4±0.5	0.4±0.4	0.4±0.3	0.39	0.5±0.5	0.4±0.3	0.3±0.3	<0.001	0.5±0.5	0.4±0.3	0.4±0.4	<0.001	0.4±0.4
Legumes, serving/d	0.2±0.2	0.4±0.3	0.7±0.8	<0.001	0.4±0.5	0.5±0.6	0.5±0.4	<0.001	0.3±0.4	0.5±0.5	0.6±0.7	<0.001	0.5±0.6
Nuts, serving/d	0.4±0.6	0.7±0.9	1.2±1.2	<0.001	0.6±0.9	0.8±1.0	0.9±0.9	<0.001	0.5±0.8	0.7±0.9	0.9±1.1	<0.001	0.7±0.9
Sweetened beverages, serving/d	1.7±1.6	1.8±1.3	1.7±1.3	0.009	1.9±1.6	1.7±1.4	1.5±0.9	<0.001	2.3±1.6	1.6±1.2	1.2±1.0	<0.001	1.8±1.4

SD, Standard deviation; AHEI-P, Alternate Healthy Eating Index for pregnancy; aMED, Alternate Mediterranean Diet; DASH, Dietary Approaches to Stop Hypertension; SFA, Saturated fatty acids; MUFA, Monounsaturated fatty acids; PUFA, polyunsaturated fatty acids; E%, % of total energy/day

^1^Values are mean±SD

^2^Diet score range for AHEI-P: Tertile 1: 27–59, Tertile 2: 60–65, Tertile 3: 66–85; aMED: Tertile 1: 0–3, Tertile 2: 4–5, Tertile 3: 6–8; DASH: Tertile 1: 11–21, Tertile 2: 22–24, Tertile 3: 25–37.

In the energy-adjusted logistic regression model 1 (Table 3), women with higher intake of PUFA, fiber, vegetables, fruits, legumes, and nuts and lower intake of SFA, red meat, and sweetened beverages had significantly higher odds of adherence to all three dietary patterns (p<0.05). Associations between intakes of nutrients and food groups with odds of adherence to the dietary patterns differed significantly for total fat (only associated with lower odds of adherence to AHEI-P, [OR: 0.96; 95% CI: 0.94–0.98], p<0.001), MUFA (only associated with higher odds of adherence to aMED, [OR: 1.06; 95% CI: 1.02–1.10], p<0.05), dairy (associated with lower odds of adherence to AHEI-P and aMED, [OR: 0.89; 95% CI: 0.84–0.95], [OR: 0.86; 95% CI: 0.81–0.91], respectively, p<0.001, but with higher odds of adherence to DASH, [OR: 1.10; 95% CI: 1.04–1.17], p<0.05), whole grain (associated with higher odds of adherence to aMED and DASH, [OR: 1.10; 95% CI: 1.04–1.17], p<0.001, but not AHEI-P) and fish (associated with higher odds of adherence to AHEI-P, [OR: 1.36; 95% CI: 1.02–1.80], p<0.05 and aMED, [OR: 1.79; 95% CI: 1.35–2.38], p<0.001, but with lower odds of adherence to DASH, [OR: 0.67; 95% CI: 0.52–0.86], p<0.05). Similar results, as in the energy adjusted model 1, were observed in the adjusted model 2 for all three dietary patterns, except for the AHEI-P. For AHEI-P the associations with PUFA, fish and sweetened beverages were not significant (Table 3).

**Table 3:** Multivariable regression analysis of the associations between maternal dietary intake and adherence to the AHEI-P, aMED and DASH dietary patterns indices.

Maternal dietary intake	AHEI-P				aMED				DASH			
	Model 1^1^		Model 2^2^		Model 1^1^		Model 2^2^		Model 1^1^		Model 2^2^	
	OR	95% CI	OR	95% CI	OR	95% CI	OR	95% CI	OR	95% CI	OR	95% CI
Carbohydrate, E%	1.04	1.02–1.05**	1.04	1.03–1.06**	1.01	0.99–1.02	1.01	0.99–1.03	1.01	1.00–1.03*	1.02	1.00–1.03*
Protein, E%	1.00	0.97–1.04	0.99	0.94–1.03	1.01	0.97–1.04	0.99	0.96–1.04	0.98	0.95–1.02	0.96	0.93–1.00
Fat, E%	0.96	0.94–0.98**	0.95	0.93–0.97**	1.00	0.98–1.02	0.99	0.98–1.02	1.00	0.99–1.02	1.00	0.98–1.02
PUFA, E%	1.07	1.01–1.13*	1.05	0.99–1.11	1.24	1.17–1.32**	1.23	1.15–1.32**	1.13	1.07–1.19**	1.12	1.06–1.19**
MUFA, E%	0.97	0.93–1.01	0.96	0.92–1.00	1.06	1.02–1.10*	1.05	1.01–1.09*	1.02	0.99–1.06	1.02	0.98–1.06
SFA, E%	0.78	0.74–0.82**	0.78	0.73–0.83**	0.82	0.78–0.86**	0.81	0.77–0.86**	0.92	0.88–0.96**	0.93	0.89–0.97**
Fiber, g/d	1.17	1.14–1.19**	1.16	1.13–1.19**	3.41	2.76–4.23**	1.12	1.09–1.15**	1.15	1.12–1.17**	1.15	1.12–1.17**
Vegetables, serving/d	2.81	2.35–3.37**	2.69	2.22–3.28**	1.41	1.25–1.58**	1.28	1.14–1.45**	1.63	1.44–1.84**	1.52	1.34–1.74**
Fruits, serving/d	1.19	1.14–1.26**	1.20	1.14–1.27**	1.09	1.04–1.13**	1.09	1.05–1.14**	1.19	1.14–1.25**	1.19	1.14–1.25**
Dairy, serving/d	0.89	0.84–0.95**	0.89	0.84–0.96**	0.86	0.81–0.91**	0.85	0.79–0.91**	1.10	1.04–1.17*	1.11	1.04–1.18*
Wholegrain, serving/d	1.29	0.98–1.71	1.12	0.84–1.49	2.19	1.61–2.99**	1.92	1.38–2.67**	4.27	3.04–5.99**	4.09	2.83–5.92**
Fish, serving/d	1.36	1.02–1.80*	1.16	0.87–1.56	1.79	1.35–2.38**	1.55	1.15–2.07*	0.67	0.52–0.86*	0.58	0.43–0.77**
Red meat, serving/d	0.25	0.16–0.38**	0.16	0.09–0.27**	0.56	0.39–0.79*	0.47	0.31–0.70**	0.24	0.16–0.36**	0.16	0.09-.025**
Legumes, serving/d	8.38	5.17–13.59**	7.55	4.51–12.64**	4.56	3.00–6.92**	3.49	2.29–5.35**	2.34	1.68–3.28**	2.01	1.42–2.83**
Nuts, serving/d	1.35	1.13–1.60**	1.32	1.09–1.60*	1.96	1.63–2.35**	2.01	1.64–2.47**	1.69	1.43–1.99**	1.71	1.42–2.05**
Sweetened beverages, serving/d	0.89	0.81–0.98*	0.91	0.83–1.01	0.88	0.80–0.96*	0.88	0.80–0.97*	0.49	0.43–0.56**	0.49	0.42–0.56**

CI, Confidence interval; OR, odds ratio; AHEI-P, Alternate Healthy Eating Index for pregnancy; aMED, Alternate Mediterranean Diet; DASH, Dietary Approaches to Stop Hypertension SFA, Saturated fatty acids; MUFA, Monounsaturated fatty acids; PUFA, polyunsaturated fatty acids; E%, % of total energy/day

^1^Adjusted for energy intake

^2^Adjusted for energy intake, age, gravidity, gestational age, education level and occupation status

*p-value less than 0.05

**p-value <0.001

## Discussion

In this prospective cohort study, adherence to three different dietary patterns and their associations with dietary intake among pregnant women in the UAE was studied. The three dietary indices were moderately correlated, despite having many similarities in how they were scored, and the dietary components included. There were also some differences in dietary profile between the three indices, which indicates that they capture different aspects of dietary intake.

Our study evaluated adherence to several healthy dietary patterns, sharing several common components, and found they were moderately correlated, suggesting a common but perhaps somewhat differential impact on health. Food components considered healthy in all three dietary indices were vegetables, fruits, nuts, legumes, and whole grains, and unhealthy components were red and processed meat. However, there are some differences in which dietary components were included in the indices, such as fiber, white meat, fish, low-fat dairy, sweetened beverages, and nutrients such as trans fatty acids, calcium, folate, iron, sodium, PUFA/SFA ratio, MUFA/SFA ratio. Intake of many nutrients and food groups were similarly related to the three dietary indices; however, there were also some differences, for example, in total energy intake, fat, MUFA, whole grain, dairy, and fish. The AHEI-P score assesses adherence to the 2010 USDA Dietary Guidelines for Americans with modifications for pregnancy [37], and scores are made based on pre-specified intake levels. The aMED [10, 12], and DASH [14] assess adherence to dietary patterns relative to the medians or quantiles intake within the study sample. These differences in scoring may have contributed to the differences in terms of nutrient profiles. In addition, the scores include different dietary components, which likely also explain why intake of nutrients and food groups differ between the indices. For example, intake of MUFA is only included in the scoring of aMED and was subsequently only associated with higher adherence to aMED. Fish intake was included in the scoring of both AHEI-P (defined as white meat) and aMED but not in DASH, which probably explains the positive association with AHEI and aMED only. On the other hand, total fat intake was not included in the scoring of either index, but higher fat intake was still associated with lower odds of adherence to AHEI-P. Dairy is not included in the scoring of AHEI-P or aMED, but higher dairy intake was negatively associated with adherence to both dietary patterns, possibly due to high SFA fat content [47, 48]. In contrast, low-fat dairy intake contributes positively to the scoring of the DASH index, and higher total dairy intake was associated with higher odds of adherence to DASH. Overall, these results indicate that the three dietary patterns reflect slightly different aspects of dietary intake and cannot be used interchangeably.

Among the pregnant women in the current study, both AHEI-P and aMED captured dietary quality from an overall perspective. In terms of fat quality, all three indices capture the most important aspects; intake of SFA and PUFA. However, only AHEI-P captured fat quantity (i.e. total fat intake). In terms of carbohydrate quality, both aMED and DASH captured whole grain intake which AHEI-P did not. Intake of fiber and sugar-sweetened beverages was however captured by all indices. High adherence to the DASH index was notably associated with a higher intake of overall dairy and lower intake of fish, in direct contrast to AHEI-P and aMED. Lastly, we used both medians and tertiles of intakes to allow for a better assessment of the variations of intake within the cohort, and to have a wider range of scores instead of using the median intake level alone [49], and this also affected the associations.

The total scores for the dietary indices used in the current study weigh all included components equally, which might not be appropriate during pregnancy as some food or nutrients might be more relevant for optimal health, whereas others may be less relevant than in the non-pregnant state [50]. Furthermore, behaviors can change over time in relation to pregnancy duration [51], and women appear to improve their dietary quality during the pregnancy period [52], driven by an increased motivation for healthier dietary choices to positively influence the baby’s health [53]. Moreover, pregnant women consider following an appropriate diet as one of their most important learning needs during pregnancy [54]. However, healthy eating during pregnancy can be challenging due to various barriers experienced by women, including food aversions, cravings, nausea, vomiting, fatigue, constipation, hemorrhoids, and heartburn [55].

Dietary patterns differ between geographical locations, populations, and cultural traditions [56]. The UAE has a multiethnic population, which includes individuals originally from Mediterranean countries. This diversity has led to the influence of the Mediterranean diet within the country [57], resulting in a culinary scene where foods and spices from both the Mediterranean and Middle East converge [58, 59]. A recent study among healthy adult males and females identified a traditional dietary pattern, endogenous to the Emirati population, consisting of mixed dishes (consisting primarily of rice, red meat and poultry), vegetables and fruits, and whole milk and dairy [60]. In the UAE, no national dietary guidelines for pregnant women exist. However, the USDA Dietary Guidelines for Americans 2015–2020 are used as the main source of dietary recommendations in the UAE and have been used as a reference in developing a national food-based dietary recommendations endorsed by the Minister of Health and Prevention in the UAE and promoted by the Food and Agriculture Organization of the United Nations [61]. Considering the influence of the Mediterranean Diet on UAE dietary culture and the use of the Dietary Guidelines for Americans in the UAE, both aMED and AHEI-P are likely highly relevant when assessing adherence to a healthy diet among pregnant women in the UAE.

The findings of this study show that different dietary patterns capture unique aspects of dietary intake among pregnant women, with important public health implications. Dietary indices are commonly used in nutritional epidemiology to assess overall diet quality and are used for the same purpose; to define adherence to a healthy diet. However, our results indicate that these indices capture different dimensions of a healthy diet and are therefore not measuring the same thing. This variation is relevant for public health because it affects the data underlying dietary recommendations. Adherence to a healthy diet pattern during pregnancy reduces risks of maternal and fetal complications [30], but the variation in nutritional profile between these indices show that researchers must be thoughtful in choosing which index to use in a given setting. Understanding of how different indices capture different aspects of diet can help inform research of diet-health-relationships and by extension, inform future dietary guidelines.

### Strength and limitations

To our knowledge, this is the first study conducted in the UAE that investigates adherence to different dietary patterns and their relation to dietary intake among pregnant women. Our study has several strengths, including a relatively large sample size and the use of a validated FFQ among pregnant women in the UAE, which is a scarcely studied population. The FFQ used in this study has been recently validated among a subsample of pregnant women from the same cohort (the Mutaba’ah Study) and showed acceptable validity for most nutrients and foods [45]. However, the estimated intake of some specific nutrients was inaccurate, especially at the individual level, so some results in the current paper should be interpreted with this in mind (e.g., SFA, fish, red meat, legumes, sodium, and calcium [45]). Moreover, the results have been presented for three dietary indices, rather than using a data-driven approach, which allows a better comparability with other studies. When interpreting the current results, it is essential to also acknowledge certain limitations. There is a risk for recall bias when using self-reported questionnaires such as an FFQ. Self-reported data have however been widely utilized in epidemiological studies, demonstrating strong consistency and validity in predicting various outcomes [62]. The FFQ data were collected under the supervision of a registered dietician who was available when clarification was requested by the women to minimize missing data and facilitate accurate reporting. However, this may have contributed to additional misreporting due to social desirability bias. Moreover, as mentioned above, the validity of some nutrients was poor and should be interpreted cautiously [45]. Lastly, we have performed multiple statistical tests without correcting for multiple testing, and the results should be interpreted with this in mind.

## Conclusions

In conclusion, in this study of pregnant women in the UAE, adherence to three healthy dietary patterns (AHEI-P, aMED or DASH) was assessed. The three dietary indices correlated but captured different aspects of dietary intake. These results show that dietary indices that assess adherence to healthy dietary patterns cannot be used interchangeably, and the choice of which dietary index to use in a given study should be made with this in mind.

## Supporting information

S1 Appendix Scoring criteria for diet-quality indices. (PDF)

S1 Table Mean intake of AHEI-P score components during pregnancy. (PDF)

S2 Table Mean intake of aMED and DASH scores components during pregnancy. (PDF)
