# Supplementary material for: Dietary patterns during pregnancy in relation to maternal dietary intake: The Mutaba’ah Study
Source: PLoS One. 2024 Oct 22;19(10):e0312442. doi: 10.1371/journal.pone.0312442 (PMC11495628; doi:10.1371/journal.pone.0312442)
Supplement: S1 Appendix — (PDF) [file pone.0312442.s001.pdf]

S1 Appendix. Scoring criteria for diet-quality indices.

**Alternative Healthy Eating Index for Pregnancy (AHEI-P)[1]**					
**Components, units**	**Foods included**	**Minimum score**	**Maximum score**	**Criteria for Minimum score of 0**	**Criteria for Maximum score of 10**
Vegetables, servings/d	All vegetables (raw, cooked), tomato products, yams, legumes, excluding potatoes	0	10	0	5 servings per day
Fruit, servings/d	All fruits (raw, dried, canned) and 100% fruit juices	0	10	0	4 servings per day
Ratio of white to red meat, g/d	White meat: poultry or fish<br>Red meat: beef or lamb and processed meat	0	10	0	≥4:1
Fiber, g/d	-	0	10	0	25 gram per day
Trans fat, E%	-	0	10	≥4% of total energy	≤0.5% of total energy
Poly-to saturated fatty acids, g/d	-	0	10	≤0.1	≥1
Calcium, mg/d	-	0	10	0	≥1200 mg per day
Folate, µg/d	-	0	10	0	≥600 gram per day
Iron, mg/d	-	0	10	0	≥27 mg per day

**Alternate Mediterranean Diet (aMED)[2,3]**				
**Components, units**	**Foods included**	**Minimum score**	**Maximum score**	**Criteria for Maximum score of 1**
Vegetables (g/d)	All vegetables except potatoes	0	1	Greater than median intake (servings/d)
Fruits (g/d)	All fruit and juices	0	1	Greater than median intake (servings/d)
Legumes (g/d)	Tofu, string beans, peas, beans	0	1	Greater than median intake (servings/d)
Nuts (g/d)	Nuts, peanut butter	0	1	Greater than median intake (servings/d)

Whole grain (g/d)	Whole-grain ready-to-eat cereals, cooked cereals, crackers, dark breads, brown rice, other grains, wheat germ, bran	0	1	Greater than median intake (servings/d)
Red and processed meat (g/d)	Hot dogs, deli meat, hamburger, beef and processed meat	1	0	Less than median intake (servings/d)
Fish (g/d)	Fish and shrimp, breaded fish	0	1	Greater than median intake (servings/d)
Mono- to saturated fatty acids (g/d)	-	0	1	Greater than median intake (servings/d)
**Dietary Approaches to Stop Hypertension (DASH)[4]**				
**Components, units**	**Foods included**	**Scoring criteria**	**Q1, servings/d**	**Q5, servings/d**
Vegetables, servings/d	All vegetables except potatoes and legumes	Q1= 1 point<br>Q2 = 2 points<br>Q3= 3 points<br>Q4= 4 points<br>Q5= 5 points	1.1	4.6
Fruits, servings/d	All fruit and juices		0.7	4.1
Nuts and legumes, servings/d	Nuts and peanut butter, dried beans, peas, tofu		0.3	1.5
Whole grain, servings/d	Whole-grain ready-to-eat cereals, cooked cereals, crackers, dark breads, brown rice, other grains, wheat germ, bran		0.1	2.4
Low-fat dairy, servings/d	Skim milk, yogurt, cottage cheese		0.1	2.3
		Reverse scoring:		
Red and processed meat, servings/d	Hot dogs, deli meat, hamburger, beef and processed meat	Q1= 5 point<br>Q2 = 4 points<br>Q3= 3 points<br>Q4= 2 points<br>Q5= 1 points	0.4	1.8
Sweetened beverages, servings/d	Carbonated and noncarbonated sweetened beverages		0	1.2
Sodium, mg/d	-		1041	2676

References:

1. Rifas-Shiman SL, Rich-Edwards JW, Kleinman KP, Oken E, Gillman MW. Dietary quality during pregnancy varies by maternal characteristics in Project Viva: a US cohort. J Am Diet Assoc. 2009;109(6):1004-11. Epub 2009/05/26. doi: 10.1016/j.jada.2009.03.001. PMID: 19465182.

2. Trichopoulou A, Costacou T, Bamia C, Trichopoulos D. Adherence to a Mediterranean diet and survival in a Greek population. N Engl J Med. 2003;348(26):2599-608. Epub 2003/06/27. doi: 10.1056/NEJMoa025039. PMID: 12826634.

3. Fung TT, Rexrode KM, Mantzoros CS, Manson JE, Willett WC, Hu FB. Mediterranean diet and incidence of and mortality from coronary heart disease and stroke in women. Circulation. 2009;119(8):1093-100. Epub 2009/02/18. doi: 10.1161/circulationaha.108.816736. PMID: 19221219.

4. Fung TT, Chiuve SE, McCullough ML, Rexrode KM, Logroscino G, Hu FB. Adherence to a DASH-style diet and risk of coronary heart disease and stroke in women. Arch Intern Med. 2008;168(7):713-20. Epub 2008/04/17. doi: 10.1001/archinte.168.7.713. PMID: 18413553.
