# Supplementary material for: Dietary patterns during pregnancy in relation to maternal dietary intake: The Mutaba’ah Study
Source: PLoS One. 2024 Oct 22;19(10):e0312442. doi: 10.1371/journal.pone.0312442 (PMC11495628; doi:10.1371/journal.pone.0312442)
Supplement: S1 Table — (PDF) [file pone.0312442.s002.pdf]

S1 Table. Mean intake of AHEI-P score components during pregnancy (n=1122)

Component	Reported intake Median (p25th-p75th)	AHEI-P score Median (p25th-p75th)	Recommended intakes during pregnancy* Intake/d
Vegetables (cups/day)	1.6 (0.9-2.5)	3 (2-5)	3 cups equivalents /day
Fruit (cups/day)	4.4 (2.4-6.8)	10 (6-10)	2 cups equivalents /day
Ratio of white to red meat (g/d)	2.6 (1.5-5.7)	6 (4-10)	No recommendation**
Fiber (g/d)	26.5 (18.1-37.6)	10 (7-10)	25 g
Trans fat (E%)	0.3 (0.2-0.4)	10 (10-10)	Less than 1 E%
Ratio of PUFA to SFA (g/d)	0.6 (0.5-0.7)	5 (4-6)	No recommendation**
Calcium (mg/d)	775 (665-941)	6 (5-8)	1000 mg/d
Folate (µg/d)	496 (436-557)	8 (7-9)	600 µg/d
Iron (mg/d)	15.8 (14.2-17.5)	6 (5-6)	27 mg/d
Total score		63 (58-67)	

AHEI-P, Alternate Healthy Eating Index for pregnancy; E%, Energy percent; SFA, Saturated fatty acids; MUFA, Monounsaturated fatty acids; PUFA, polyunsaturated fatty acids; SD, Standard deviation; p25th-p75th, 25th percentile- 75th percentile.

*Recommended intake during pregnancy according to the Dietary guidelines for Americans 2020-2025 [27] among pregnant women with calorie level of pattern of 2200 kcal (mean energy intake among our pregnant women (2239 kcal per day)) and using the mean age of our pregnant women (31 years old).

** Ratio intakes are not directly comparable to the recommendations.
