# Supplementary material for: Dietary patterns during pregnancy in relation to maternal dietary intake: The Mutaba’ah Study
Source: PLoS One. 2024 Oct 22;19(10):e0312442. doi: 10.1371/journal.pone.0312442 (PMC11495628; doi:10.1371/journal.pone.0312442)
Supplement: S2 Table — (PDF) [file pone.0312442.s003.pdf]

S2 Table. Mean intake of aMED and DASH scores components during pregnancy (n=1122)

Reported intake (aMED)[a]		Reported intake (DASH)	
**Component**	**Median ($p25^{th}$-$p75^{th}$)**	**Component**	**Median ($p25^{th}$-$p75^{th}$)**
Vegetables (g/d)	95.5 (53.9-145.8)	Vegetables (servings/d)	1.1 (0.6-1.9)
Fruits (g/d)	425.0 (268.4-642.4)	Fruits (servings/d)	4.4 (2.4-6.8)
Legumes (g/d)	18.2 (7.4-31.5)	Nuts and legumes (servings/d)	0.8 (0.4-1.4)
Nuts (g/d)	21.6 (7.1-40.0)	Whole grain (servings/d)	0.3 (0.1-0.6)
Whole grain (g/d)	18.1 (7.8-37.2)	Low-fat dairy (servings/d)	0 (0-0.4)
Red and processed meat (g/d)*	27.3 (13.0-45.2)	Red and processed meat (servings/d)*	0.4 (0.1-0.6)
Fish (g/d)	32 (15.5-55.1)	Sweetened beverages (servings/d)*	1.6 (0.9-2.3)
Mono- to saturated fatty acids (g/d)	1.1 (0.9-1.5)	Sodium (mg/d)*	2133.4 (1573.8-2833.7)
Total score	4 (3-5)	Total score	23 (20-25)

aMED: Alternate Mediterranean Diet. DASH: Dietary Approaches to Stop Hypertension SD: Standard deviation . p25th-p75th: 25th percentile- 75th percentile.

[a] energy adjusted to 2000 kcal/d

*a higher score was assigned to a lower intake.
